# Increased Fecal *Lactobacillus* Is Associated With a Positive Glucose Hydrogen Breath Test in Bangladeshi Children

**DOI:** 10.1093/ofid/ofz266

**Published:** 2019-06-01

**Authors:** Jeffrey R Donowitz, Hardik I Parikh, Mami Taniuchi, Carol A Gilchrist, Rashidul Haque, Beth D Kirkpatrick, Masud Alam, Shahria Hafiz Kakon, Bushra Zarin Islam, Sajia Afreen, Mamun Kabir, Uma Nayak, E Ross Colgate, Marya P Carmolli, William A Petri

**Affiliations:** 1 Division of Pediatric Infectious Diseases, Children’s Hospital of Richmond at Virginia Commonwealth University, Richmond, Virginia; 2 Division of Infectious Diseases and International Health, University of Virginia, Charlottesville, Virginia; 3 School of Medicine Research Computing, University of Virginia, Charlottesville, Virginia; 4 Division of Parasitology, International Centre for Diarrhoeal Disease Research, Bangladesh, Dhaka, Bangladesh; 5 Department of Microbiology and Molecular Genetics, and Vaccine Testing Center, University of Vermont College of Medicine, Burlington, Vermont; 6 James P. Grant School of Public Health, Brac University, Dhaka, Bangladesh; 7 Center for Public Health Genomics, University of Virginia, Charlottesville, Charlottesville, Virginia

**Keywords:** environmental enteric dysfunction, fecal microbiome, hydrogen breath testing, *Lactobacillus*, small intestine bacterial overgrowth

## Abstract

**Background:**

Glucose hydrogen breath testing is a noninvasive test for small intestine bacterial overgrowth (SIBO). A positive glucose hydrogen breath test is common in children from low-income countries and has been found to be associated with malnutrition as measured by stunted growth. The microbiome associated with positive breath testing is relatively unstudied.

**Methods:**

We performed 16 S V4 rDNA microbiome analysis on the stool of 90 Bangladeshi children aged 2 years from an impoverished neighborhood who were tested at the same time for SIBO by glucose hydrogen breath testing. Data were analyzed by linear discriminant analysis effect size with SIBO as the outcome. Any selected genera were tested individually by Wilcoxon’s rank-sum test to ensure that linear discriminant analysis effect size results were not outlier-skewed.

**Results:**

Linear discriminant analysis effect size analysis identified *Lactobacillus* (linear discriminate analysis score, 4.59; *P* = .03) as over-represented in 15 out of the 90 children who were SIBO positive.

**Conclusions:**

These results suggest that glucose hydrogen breath test positivity in children from low-income settings may be due to an upper intestinal *Lactobacillus* bloom, potentially explaining the association of SIBO with the gut damage and inflammation that leads to malnutrition.

Small intestine bacterial overgrowth (SIBO) was first described by Barker and Hummel in 1939 when they observed macrocytic anemia in patients with small intestine structural abnormalities (strictures and anastomoses) [1]. Based on their initial observations, others went on to describe the presence of SIBO in blind loop anatomy, gastrointestinal motility disorders, and hypochlorhydria [2]. More recently, SIBO has been described in children living in impoverished settings in low-income countries [3–7]. SIBO in high-income settings has been associated with aberrant absorption of nutrients via several mechanisms, which include bacterial utilization of carbohydrates before host absorption, bile acid deconjugation leading to steatorrhea and loss of fat-soluble vitamins, bacterial B12 utilization, and protein-losing enteropathy [2]. We demonstrated that SIBO in children from low-income countries was associated with linear growth deficits in a cross-sectional analysis, a result that has been confirmed by 2 separate groups [6–8].

SIBO is classically defined as ≥10^5^ CFU/mL of growth when endoscopically obtained upper gastrointestinal (GI) samples are plated on anaerobic and aerobic media [2]. Glucose or lactulose hydrogen breath testing has been used in both the clinical and research settings as a noninvasive marker of SIBO [9]. However, little work has been done to define which species are present in the upper GI tract of SIBO patients or which microbial populations correlate with a positive breath test. In the premolecular era, one study found that SIBO diagnosed by endoscopy was associated with increased *Streptococcus*, *Lactobacillus*, and *Escherichia* species [10]. Using quantitative polymerase chain reaction (qPCR) techniques, researchers in Brazil demonstrated higher counts of *Eubacteria* in the stool of SIBO-negative children and higher counts of *Salmonella* in stool samples from lactulose hydrogen breath test–positive children [8]. Most recently, in a study of children from sub-Saharan Africa, SIBO, as diagnosed by upper GI aspirate culture, was associated with increased upper GI abundance of oropharyngeal species, including *Haemophilus*, *Streptococcus*, *Neisseria*, *Veillonella*, *Porphyromonas*, and *Moraxella*, as determined by 16 S rDNA sequencing. Stool samples from stunted children, who had a SIBO prevalence of 91%, had a similarly high abundance of oropharyngeal flora, with *Lactobacillus salivarius* being the most significant single organism, with a 333.9-fold increase from nonstunted children [7]. The work presented here is the first to utilize 16 S rDNA analysis to define the microbial populations in stool that correspond to SIBO, as defined by a positive glucose hydrogen breath test (GHBT).

## METHODS

This work was done on samples from the Performance of Rotavirus and Oral Polio Vaccines in Developing Countries (PROVIDE) study sample bank. Briefly, PROVIDE is a longitudinal study of Bangladeshi infants with the primary objective of investigating the association between environmental enteric dysfunction (EED) and the underperformance of oral vaccines. Detailed methods of this study have been published elsewhere [11, 12]. Briefly, 700 children were enrolled within 7 days of birth. Subjects were randomized to receive the oral rotavirus vaccine (Rotarix) and/or the inactivated polio vaccine in a 2×2 design. All children received the oral polio vaccine per the Expanded Program on Immunization, Bangladesh. Rolling admission spanned from May 2011 through November 2014. Results of this study examining the association of biomarkers of EED and oral polio vaccine failure, rotavirus vaccine failure, growth through 1 year of age, the impact of enteropathogens on oral rotavirus and polio vaccination, and the association of Rotarix vaccination and serum zinc levels with severe rotavirus diarrhea have been published elsewhere [11, 13, 14]. The PROVIDE study was approved by the Institutional Review Boards at the University of Virginia and the University of Vermont and by the Ethics and Research Review Committees at the International Centre for Diarrhoeal Disease Research, Bangladesh.

Children who were assessed and had an weight-for-age Z score (WAZ) ≤–3 SD were not tested and were referred to nutritional therapy. The GHBT was compared with markers of enteric inflammation and intestinal epithelial cell damage, growth parameters, diarrheal disease, and sanitation. The methods of GHBT and these results have been previously published [6]. SIBO positivity was defined as a ≥12-ppm increase in exhaled breath hydrogen over the subject’s baseline sample.

At the 2-year study visit, stool was collected from the patients on the morning of GHBT before glucose consumption. Stool was transported from the field to our laboratory at 4°C, aliquoted in DNase- and RNase-free cryovials, and stored at –80°C on the day of collection. Samples were shipped from our laboratory in Bangladesh to our laboratory in Virginia on dry ice, where they were replaced in –80°C storage. Samples were then removed, thawed, and 200 ug was removed for total nucleic acid (TNA) extraction. Extraction was performed using a slightly modified protocol utilizing the QIAamp DNA Stool Mini Kit (Qiagen, Gaithersburg, MD), which has been described elsewhere [13, 15]. TNA was stored at –80ºC until testing. Positive extraction controls were achieved by spiking phocine herpesvirus (Erasmus MC, Department of Virology, Rotterdam, the Netherlands) and bacteriophage MS2 (ATCC 15597B; American Type Culture Collection, Manassas, VA) into each sample during the extraction process.

The entire 255-bp V4 region of the 16 S rDNA gene was amplified as previously described, using phased Illumina-eubacteria primers to amplify the V4 16 S rDNA region (515F–806R) and to add the adaptors necessary for illumina sequencing and the GOLAY index necessary for de-multiplexing after parallel sequencing [16, 17]. Negative controls included the addition of extraction blanks that were tested throughout the amplification and sequencing process to ensure they remained negative. As a positive PCR control, DNA extracted from the HM-782D Mock Bacteria Community (ATCC through BEI Resources) was run on each plate and added to the library. A PhiX DNA library was spiked into the 16S sequencing run (20%) to increase genetic diversity before parallel sequencing in both forward and reverse directions using the Miseq V3 kit and machine (per the manufacturer’s protocol).

Raw paired-end sequences were trimmed to a length of 200 bp to remove poor-quality base calls in the tail end of both forward and reverse reads. V4 rRNA amplicons were generated by merging the overlapping ends of trimmed paired-end reads using the MeFiT pipeline [18]. Amplicons with a maximum expected error as a percentage of read length (meep) score <1.0, that is, <1 error per 100 bases, were retained for further analysis. Chimeric amplicons were removed using the SILVA LTPs123 reference database [19], as implemented in VSEARCH, version 2.7.1 [20]. Operational taxonomic units (OTUs) were identified by de novo clustering pooled sequences from all samples at 97% identity threshold, using USEARCH, version 10.0.240 [21]. Taxonomic assignments of OTUs up to the genus level was performed using the Bayesian classifier Ribosomal Database Project (version 2.12) [22]. Species-level assignments were made by aligning the OTUs to the SILVA LTPs123 database [19].

Within-sample alpha diversity was measured using the Shannon index. Pairwise Bray-Curtis dissimilarity distances were calculated using the *vegan* R package [23], followed by principal coordinates analysis using the *phyloseq* R package [24]. Linear discriminant analysis effect size (LEfSe) was used to perform high-dimensional class comparisons to identify features that discriminate the phenotypes, that is, SIBO-positive and -negative groups [25].

## RESULTS

One hundred and three children were screened by GHBT for SIBO. Nine were excluded due to the weight-for-age Z score being ≤–3 SD. Three children were unable to complete the 3-hour test period, and 1 parent refused breath collection. Thus, 90 children successfully completed the GHBT. As previously reported, 15 of the children were SIBO positive and 75 were SIBO negative, based on a ≥12-ppm rise in H_2_ [6]. Enrollment characteristics did not differ significantly between the 2 groups [6]. Also as previously reported, fecal calprotectin and fecal Reg1B were elevated in the SIBO-positive children, and SIBO-positive children were more likely to live in less sanitary conditions and had increased growth stunting compared with SIBO-negative children [6].

**Figure 1.**
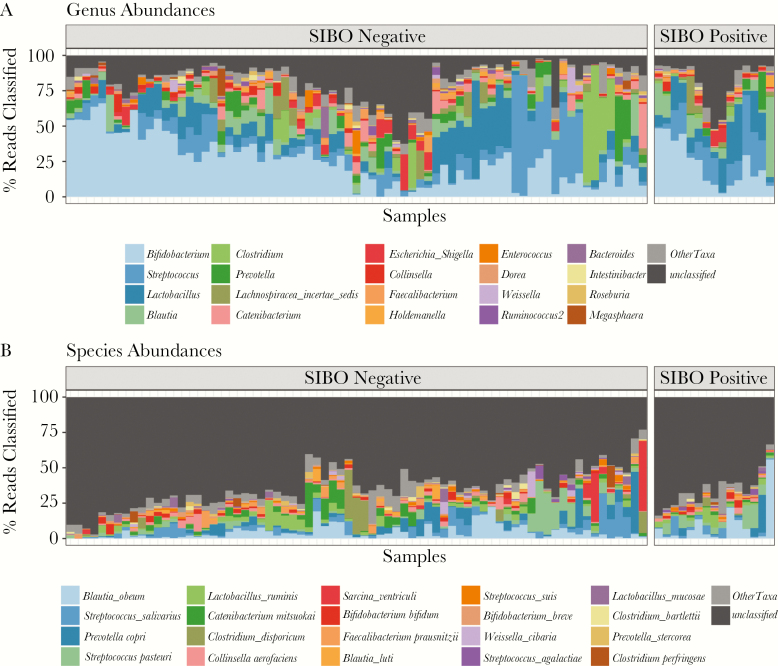
Genus- and species-level abundances. Stacked bar plot showing percentage of reads classified at the genus (A) and species (B) levels for samples grouped by small intestine bacterial overgrowth (SIBO) status. The samples are clustered based on their Bray-Curtis dissimilarity distances using the Ward method. The top 20 most abundant genera and species are plotted. The counts for the remaining classified taxa are agglomerated into “Other Taxa.” On average, 82.9% of reads/sample were classified at the genus level and 35.9% of reads/sample were classified at the species level.

**Figure 2.**
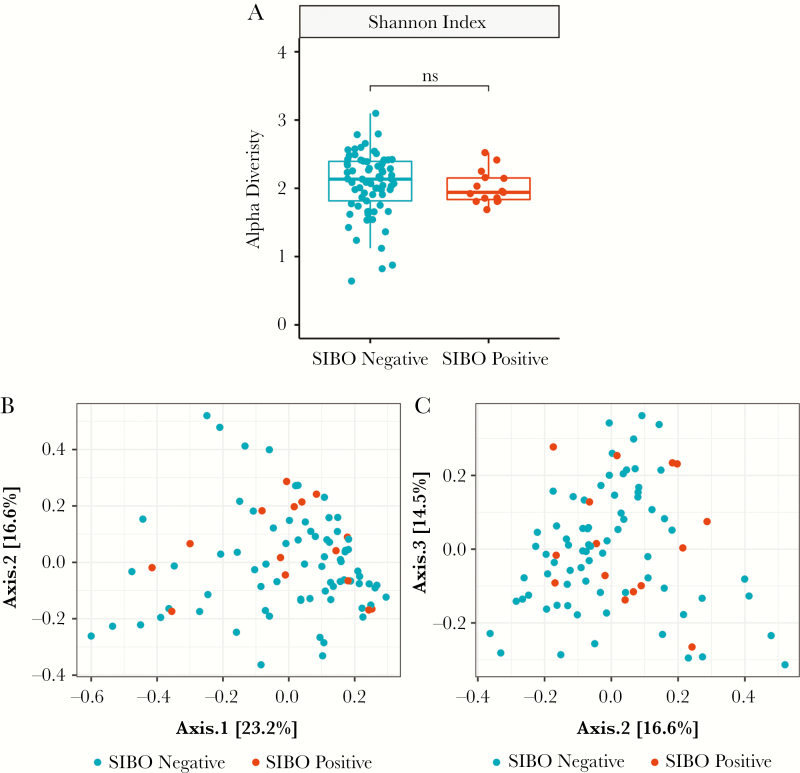
Diversity indices. A, Alpha diversity in samples by small intestine bacterial overgrowth (SIBO) status, as measured using Shannon’s index of genus-level classification, demonstrated no statistically significant difference as measured by Mann-Whitney *U* testing. B, Bray-Curtis dissimilarity between samples was calculated at genus-level classifications. Principal coordinates ordination of the distances indicated no clear shift in microbiome between SIBO statuses.

Sequencing had an average depth of 62 247 reads per sample (range, 4903–118 484 reads per sample). One thousand two hundred sixty-seven OTUs were identified from the 88 samples (2 stool samples failed to amplify, both from SIBO-negative children). Taxonomic assignment of OTUs and abundance estimations revealed variable levels of classification of reads. At the phylum and family levels, on average 97.6% and 96.8% of reads were classified per sample, respectively. However, at lower taxonomic levels, fewer reads were classified, with 82.9% and 35.9% reads on average per sample at the genus and species levels, respectively (Figure 1; Supplementary Figure 1). Thus, a decision was made to proceed with further analysis at the genus level. The Shannon alpha diversity index at the genus level, which takes into account the richness and evenness of the community, was not significantly different between SIBO-positive and SIBO-negative groups (mean, 2.01 vs 2.06; *P* = .29). Principal coordinates analysis of the Bray-Curtis dissimilarity distances at the genus level indicated no apparent shift in the community structure between SIBO-positive and SIBO-negative children (Figure 2). Ordination of phylogenetically informative distances, like unweighted and weighted UniFrac distances, also showed no apparent shifts in the microbial community (Supplementary Figure 2).

**Figure 3.**
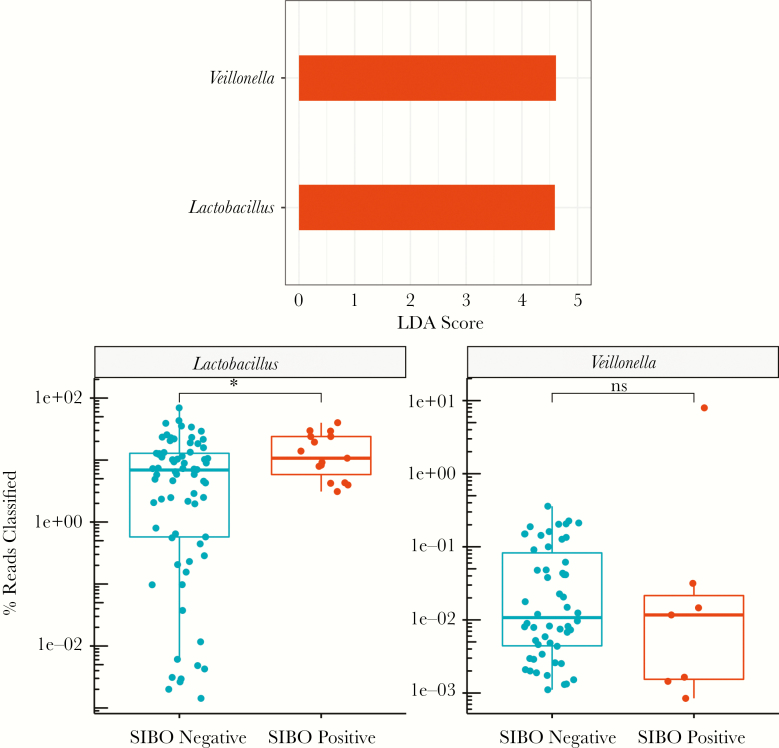
Predictive analysis. A, Linear discriminant analysis effect size selected 2 genera indicative of small intestine bacterial overgrowth (SIBO) status, *Lactobacillus* and *Veillonella*. B, Boxplots for relative abundance of *Lactobacillus* and *Veillonella* between SIBO-negative and SIBO-positive samples show a significant increase in abundance of *Lactobacillus* in SIBO-positive samples (Wilcoxon’s *P* = .028). *Veillonella* did not maintain significance (Wilcoxon’s *P* = .38).

Linear discriminant analysis effect size analysis identified 2 genera that differentiated SIBO status, *Lactobacillus* (linear discriminant analysis [LDA] score, 4.59; *P* = .03) and *Veillonella* (LDA score, 4.62; *P* = .03), both higher in the SIBO-positive group. When the relative abundance of these genera was examined individually using Wilcoxon’s rank-sum test, only the difference in *Lactobacillus* remained statistically significant (mean percentage of reads, 15.47% vs 10.09%; *P* = .03). *Veillonella* relative abundance was not significantly different (mean percentage of reads, 1.15% vs 0.06%; *P* = .38) when analyzed by Wilcoxon’s rank-sum test, suggesting that LEfSe’s identification of the taxa was likely due to a single outlier in the SIBO-positive group (Figure 3).

## DISCUSSION

The key discovery of this work was the association of an increase in fecal *Lactobacillus* species with GHBT positivity. Positive GHBT has been associated with SIBO as diagnosed by culture of upper intestinal aspirates [9]. Early studies attempting to delineate exactly which genera or species are responsible for GHBT positivity demonstrated increased growth of *Lactobacillus*, *Escherichia,* and *Streptococcal* species associated with a positive test [10]. These early studies were conducted using culture techniques before the development of current molecular analytic techniques. The use of 16 S rDNA in our study may account for the difference in our observations from the Brazilian study. This study analyzing children in Brazil by lactulose hydrogen/ methane breath testing found a higher prevalence of *Salmonella* species in the stool of breath test–positive children as measured by qPCR. *Lactobacillus* quantity, again determined by qPCR, did not differ between breath test–positive and –negative subjects [8]. The only study to investigate SIBO utilizing 16S rDNA techniques was conducted on African children who were stunted and did find *Lactobacillus* to be over-represented in the stool of stunted children. SIBO in that study, as diagnosed by semiquantitative culture of upper GI aspirates, was found in 42 of the 48 children sampled (87.5%). However, duodenal 16s rDNA analysis was not performed to directly compare SIBO-negative and -positive subjects [7]. Our work supports the findings of the African study and suggests that GHBT is detecting a similar dysbiosis to that described in detail in their analysis [7].

Recent work has shown that *Lactobacillus* overgrowth in a *Drosophila* mutant can lead to increased lactic acid production by the enteric microbiota and trigger activation of the intestinal NADPH oxidase Nox and an increase in reactive oxygen species [26]. This could explain the link between GHBT positivity and the enteric inflammation and epithelial cell damage that hallmark EED, which we showed in our previous analysis of this cohort [6]. EED is a significant cause of morbidity in the low-income pediatric population and has been associated with failure of oral vaccines, neurodevelopmental delays, and growth stunting [11, 27–29]. Furthermore, GHBT positivity was directly associated with prior linear growth stunting in this cohort [6]. Stunted children have been shown to have increased mortality by 5 years of age, and stunting in early childhood has been hypothesized to contribute to diabetes and metabolic disturbances later in life [30]. Stunting has been associated with neurodevelopmental delays that may lead to decreased productivity later in life [29, 31]. Given that one-fourth of the world’s children under 5 years of age are estimated to be stunted, understanding the contribution of SIBO to the pathogenesis of this complex phenotype could be critical to improving child health, and thus subsequently adult health and productivity in low- and middle-income countries [32].

This study has several strengths, with the most important being measurement of SIBO in children in a lower-socioeconomic urban neighborhood in Dhaka and the use of 16 S rDNA sequencing to describe the fecal microbiota of children with positive breath tests. GHBT and collection of fecal samples were accomplished on the same day, and a cold chain was preserved to ensure technical completion of DNA sequencing.

A limitation of this work was use of fecal samples instead of duodenal samples to quantify the microbiome associated with GHBT positivity. The GHBT is believed to be specific for small intestinal dysbiosis, as glucose will be completely absorbed in the upper intestine when competing bacteria are not present. The degree to which fecal bacteria are representative of those in the small bowel is unknown; however, *Lactobacillus* is generally an upper intestinal commensal. This supports our theory that changes in small bowel bacterial populations can be detected through fecal analysis. A second limitation of this work is that it was a cross-sectional study of infants. The intestinal microbiome is known to change dramatically over the first several years of life [33–35], which could potentially confound these analyses. To confirm our findings, further investigation needs to be conducted to determine if *Lactobacillus* abundance is higher in children with GHBT positivity across varying age groups.

The current understanding of environmental enteric dysfunction and the contribution of SIBO is limited. EED involves inflammatory infiltrate into the lamina propria and changes to the intestinal architecture, which include blunting of villi and crypt hypoplasia [28]. How changes to the intestinal microbiota potentiate the development of EED or result from it is poorly understood. However, if strategies are going to be designed to combat the deleterious effects of EED on children in low- and middle-income countries, the specific dysbiosis of the small intestine and how these shifts in microbial populations affect children living in unsanitary conditions must be defined.

## Supplementary Data

Supplementary materials are available at *Open Forum Infectious Diseases* online. Consisting of data provided by the authors to benefit the reader, the posted materials are not copyedited and are the sole responsibility of the authors, so questions or comments should be addressed to the corresponding author.

ofz266_suppl_supplementary_figure_s1

ofz266_suppl_supplementary_figure_s2

ofz266_suppl_supplementary_figure_legends
