# Supplementary figures and images for: Increased Fecal Lactobacillus Is Associated With a Positive Glucose Hydrogen Breath Test in Bangladeshi Children
Source: Open Forum Infect Dis. 2019 Jun 1;6(7):ofz266. doi: 10.1093/ofid/ofz266 (PMC6602902; doi:10.1093/ofid/ofz266)

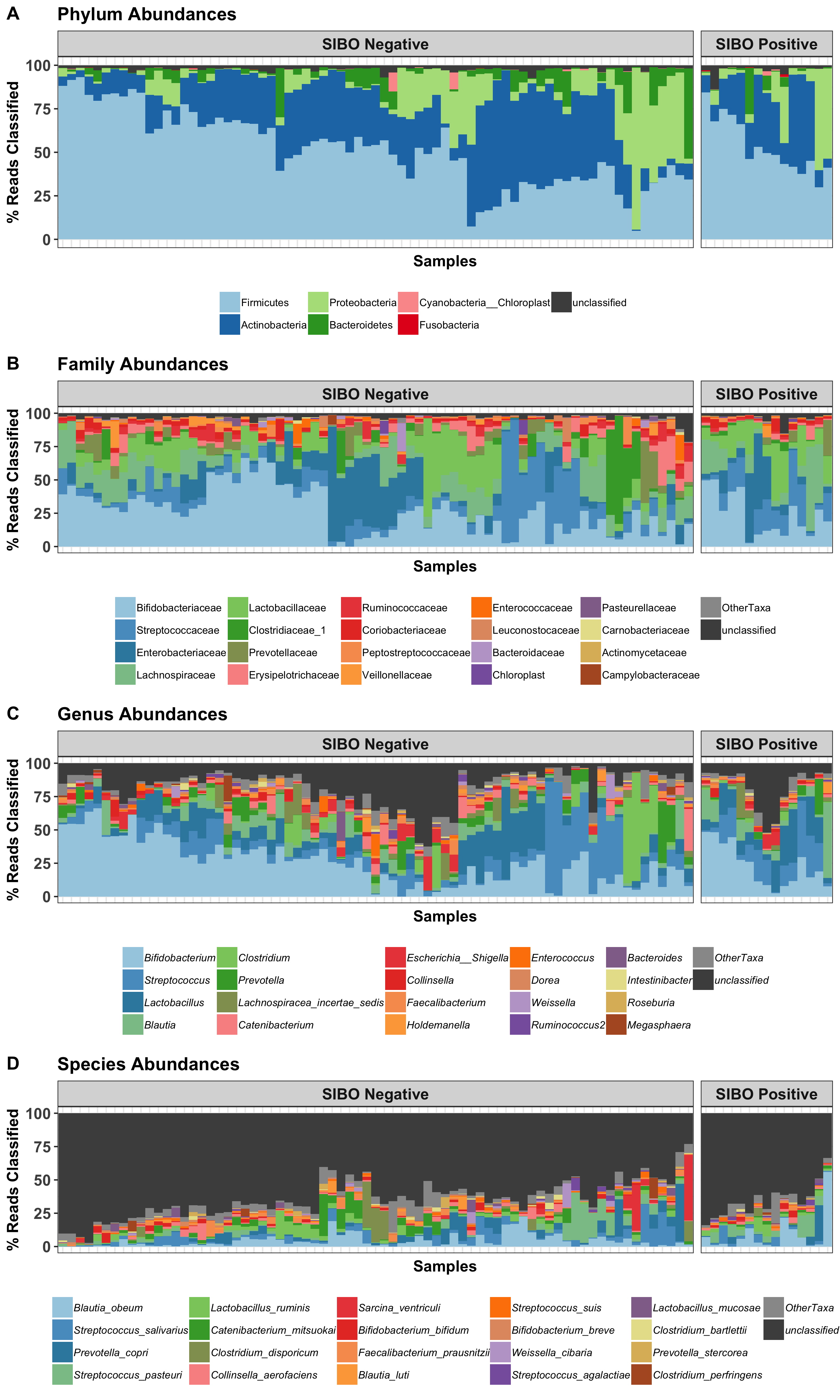

Supplement: ofz266_suppl_supplementary_figure_s1 [file ofz266_suppl_supplementary_figure_s1.png]

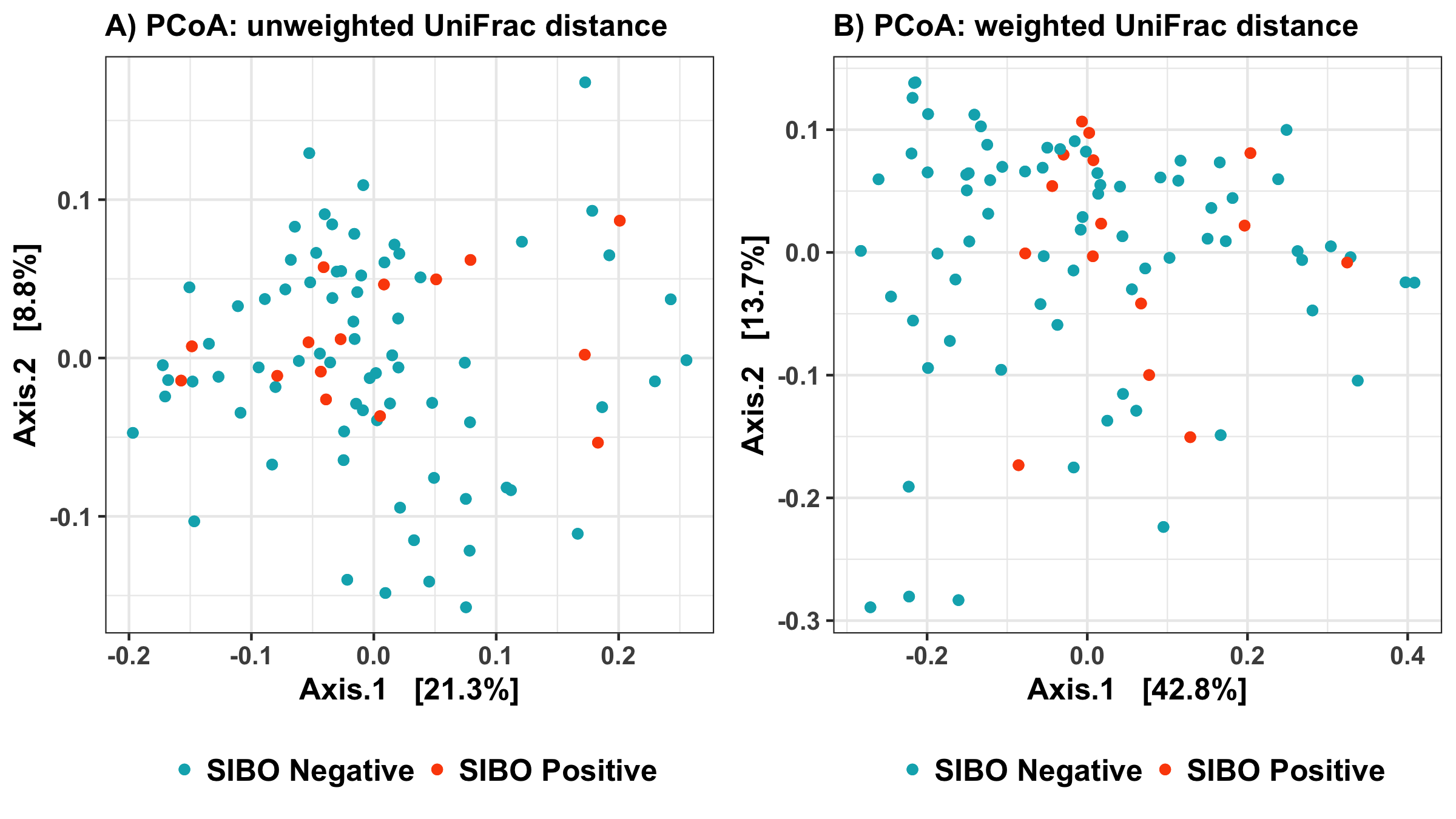

Supplement: ofz266_suppl_supplementary_figure_s2 [file ofz266_suppl_supplementary_figure_s2.png]
