# Supplementary material for: Increased Fecal Lactobacillus Is Associated With a Positive Glucose Hydrogen Breath Test in Bangladeshi Children
Source: Open Forum Infect Dis. 2019 Jun 1;6(7):ofz266. doi: 10.1093/ofid/ofz266 (PMC6602902; doi:10.1093/ofid/ofz266)
Supplement: ofz266_suppl_supplementary_figure_legends [file ofz266_suppl_supplementary_figure_legends.docx]

**Supplementary Figure Legends:**

**Figure S1: Taxonomic Abundances. Stacked bar plots showing percentage of reads classified at phylum (A), family (B), genus (C) and species (D) levels for samples grouped by SIBO status. The samples in each panel are clustered by their bray-curtis dissimilarity distances. For family, genus and species plots, the top 20 most abundant taxa are plotted. The counts for remaining classified reads are agglomerated into the “Other Taxa” category. On average 97.6%, 96.8%, 82.9% and 35.9% reads per sample are classified at phylum, family, genus and species level, respectively.**

**Figure S2: Phylogenetic Beta Diversity at Genus-level. Principal Co-ordinate Analysis of unweighted (A) and weighted (B) UniFrac distances. No apparent shifts in the community structure between samples from SIBO positive and SIBO negative children.**
